# Dermatitis linearis outbreak associated with *Paederus balcanicus* in Austria

**DOI:** 10.1007/s00508-022-02047-6

**Published:** 2022-06-20

**Authors:** Karin Bakran-Lebl, Kaan Harmankaya, Hans-Peter Fuehrer, Erich Heidenreich, Lilla Marton, Thomas Zechmeister, Franz Allerberger, Matthias Preusser

**Affiliations:** 1 Institute for Medical Microbiology and Hygiene, Austrian Agency for Health and Food Safety (AGES), Währingerstr. 25A, 1090 Vienna, Austria; 2 Palais Kinsky, Freyung 4/17, 1010 Vienna, Austria; 3 Institute of Parasitology, University of Veterinary Medicine Vienna, Vienna, Austria; 4 Center for Cancer Research, Medical University of Vienna, Vienna, Austria; 5 Biological Station Lake Neusiedl, Illmitz, Austria; 6 Division of Oncology, Department of Medicine I, Medical University of Vienna, Vienna, Austria

**Keywords:** *Paederus balcanicus*, Rove beetle, Staphylinidae, Exanthema, Lake Neusiedl

## Abstract

**Background:**

Dermatitis linearis is a toxic skin lesion caused by contact with certain beetles of the genus *Paederus* (Coleoptera: Staphylinidae). Dermatitis linearis outbreaks have been described mainly in tropical and subtropical regions, but so far not in Central Europe, and are considered an emerging public health concern potentially associated with climate change.

**Material and methods:**

Following diagnosis of dermatitis linearis in a cluster of six adults and one child with reported exposure to beetles with morphological characteristics of *Paederus* species at a recreational public open-air bath at Lake Neusiedl (Illmitz, Burgenland, Austria), we performed on-site inspection and installed light and pitfall traps. Collected beetle specimens of the genus *Paederus* were classified using morphological characteristics and DNA barcoding.

**Results:**

A total of 32 *Paederus* beetles were collected using an aspirator (*n* = 2) and light traps (*n* = 30). No individuals of the genus *Paederus* were captured with the pitfall traps. Morphological analyses identified them as members of the *Paederus balcanicus *species, which was confirmed by genetic specification of four arbitrarily chosen individuals. Dermatitis linearis lesions were treated with topical steroids and healed but partly leaving scars and hyperpigmentation, over the course of a few weeks in all affected persons.

**Conclusion:**

We report for the first time (a) an outbreak of dermatitis linearis associated with exposure to autochthonous *Paederus* species in Austria, and (b) that contact to the species *Paederus balcanicus* may cause dermatitis linearis in humans. Adequate measures should be taken to prevent dermatitis linearis outbreaks in areas with resident *Paederus *occurrence.

## Introduction

Rove beetles (family Staphylinidae) represent one of the largest groups of beetles with more than 50,000 known species worldwide. *Paederus* is a genus consisting of more than 500 species within this family. *Paederus* beetles are relatively slender and small (approximately 1.5 mm wide and 7–10 mm long) and easily recognized by the characteristic coloring (black head with red or orange thorax). They inhabit moist areas, such as marshes and edges of freshwater lakes and are active during the day but are also attracted to incandescent and fluorescent light at night. *Paederus* beetles are predators of other insects and nematodes and in some agricultural areas are considered as helpful in the biological control of crop pests; however, local *Paederus* invasion and proliferation associated with anthropogenic and landscape disturbances has become an issue in some urban settings, particularly in Africa and Southeast Asia [1].

At least 20 species within the genus *Paederus *cause characteristic skin lesions on contact. These injuries are caused by the vesicant toxin pederin, which is present in the hemolymph of female beetles [2]. Pederin is an amide with two tetrahydropyran rings produced by endosymbiotic *Pseudomonas* bacteria and prevents cell division, inhibits protein and DNA synthesis. It is among the most potent known non-proteinaceous toxins. *Paederus* beetles do not bite or sting, but the toxin is typically released when the beetles are crushed while being brushed away, thus causing characteristic linear skin irritations (dermatitis linearis or whiplash dermatitis). Interestingly, initial skin contact with pederin causes no injury, but lesions emerge later within 12–36 h after contact. Toxic hemolymph present at a primary site may be transferred to a secondary site by touch, thus causing characteristic kissing or mirror lesions. Treatment of *Paederus* lesions is ill-defined, but washing of exposed skin areas with water and soap and application of (topical) steroids and antihistamines are commonly recommended [1, 3]. The use of oral antibiotics is recommended to prevent or treat secondary infections [4, 5] but has also been reported to reduce healing time of dermatitis linearis in general [6]. Lesions usually resolve within days to weeks but may leave behind scarring and hyperpigmentation. In the eye, pederin may cause severe conjunctivitis that may lead to blindness (Nairobi eye).

Dermatitis linearis outbreaks have been described mainly in local populations in tropical and subtropical regions and in travellers [3]; however, invasion of *Paederus *spp. into human settings, potentially associated with climate change, is an emerging public health concern throughout the world. In Europe, dermatitis linearis outbreaks have so far been described anecdotally in Italy and were associated with *P. fuscipes *[7].

Here, we report for the first time a clustered outbreak of dermatitis linearis in Austria caused by a resident *Paederus *species previously unknown to have toxic properties and highlight potential implications for public health authorities.

## Material and methods

### Sample collection

Samples were collected at the sunbathing lawn in the open-air bath of Illmitz, Lake Neusiedl. At 3 locations a light trap and a pitfall trap, a black 1 l bucket filled ~2/3 with saline (200 g NaCl/1 l water) and a few drops of detergent, trap was covered with a coarse-meshed grid, were set up. The first location (A) was set up directly near the location of the reported incident (47.7536°N, 16.7399°E, about 30 m from the waterline), the second location (B) was set up about 75 m apart on the sunbathing lawn (47.7541°N, 16.740°E, about 30 m from the waterline), the third location (C) was at the end of the sunbathing lawn and at the beginning of the reed belt (47.7548°N, 16.7428°E, about 150 m from the waterline of the lake). Traps were set up at 20:00 on 15 June 2021 and were collected the following day at 07:00.

### Sample classification

Collected specimens of the genus *Paederus* were identified to the species level using morphological characteristics following the key of Lompe [8]. To confirm the morphological identification four arbitrarily chosen individuals (one from the aspirator collection and one from each of the light traps) were analyzed by DNA barcoding. The DNA was extracted from one leg of each individual and three 1.4 mm ceramic beads (Precellys Ceramic Kit 2.8 mm/1.4 mm, Peqlab, Erlangen, Germany) were added to each tissue sample. Homogenization was performed with a TyssueLyser II (Qiagen, Hilden, Germany). Afterwards DNA was extracted using the DNeasy® Blood and Tissue kit according to the manufacturer’s instructions (Qiagen). Conventional polymerase chain reactions (PCRs), targeting the barcode region within the mitochondrial cytochrome c oxidase subunit I gene (COI) using primers LCO1490 and HCO2198 were performed as reported previously [9]. PCR products were sequenced at LGC Genomics GmbH, Berlin, Germany. Resulting sequences were compared to sequences available on GenBank® and BOLD systems databases. Aligned sequences were uploaded to GenBank® (MZ563430, MZ563432-MZ563434).

## Results

### Outbreak cluster and clinical presentation

**Fig. 1:**
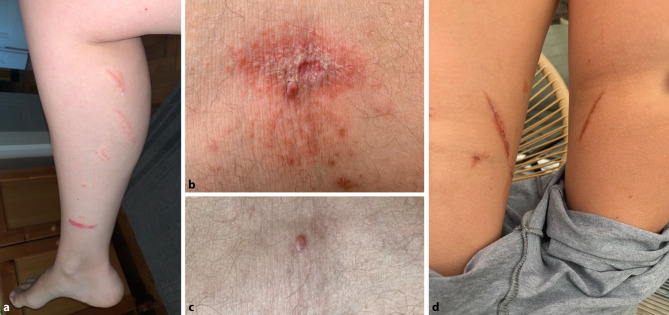
**a** Dermatitis linearis lesions on the calf of a woman 4 days after exposure to *Paederus balcanicus*. **b** Papulovesicular exanthema with superficial necrosis in the back of the knee of a man 9 days after exposure to *Paederus balcanicus. ***c** Follow-up picture of the lesion shown in B showing slight residual hyperpigmentation 7 months after beetle exposure. **d** Mirror lesions on the inner thighs of a 9-year-old child 9 days after exposure to *Paederus balcanicus*

On 3 June 2021, a group of 15 family members (7 children, 8 adults) spent an afternoon at a recreational public open-air bath at Lake Neusiedl (Illmitz, Burgenland, Austria). Soon after arrival, they noticed a swarm of small and slender insects with black and red-colored bodies, which crawled in the grass and on their possessions (picnic sets and blankets). The insects also crawled on the bodies and extremities of some of the persons; however, no stings or bites were reported. The persons reported that they had brushed the insects off the skin. In the late afternoon of 4 June 2021 one of the adults developed mostly linear papulovesicular exanthema below the right eye, on the nose, and the left shoulder and sought medical advice. Physical examinations, detailed anamnesis and skin inspections were performed by a board-certified dermatologist and a board-certified internal medicine specialist. Over the course of several days, an additional number of six family members (1 child aged 9 years and 5 adults) developed similar skin irritations with characteristic linear morphology on the arms and legs (Fig. 1) including formation of mirror lesions. Table 1 summarizes the clinical characteristics of the seven persons with skin lesions attributed to *Paederus balcanicus *exposure.

**Table 1:** Clinical characteristics of seven patients with skin lesions attributed to *Paederus balcanicus exposure*

Patient ID	Age (years)	Gender	Localization of skin affections	Time (days) of onset after beetle exposure	Duration of florid skin lesion
1	44	m	Back of the right knee	8	3 weeks
2	44	f	Face, right shoulder, both legs	1	3 weeks
3	39	f	Both calves	4	3 weeks
4	44	m	Abdomen	6	3 weeks
5	9	f	Both inner thighs	3	2 weeks
6	45	m	Both forearms	7	2 weeks
7	77	m	Both legs	7	2 weeks

All lesions were rapidly treated after onset with topical methylprednisolone aceponate ointment 0.1%. Despite this treatment, most of the individual lesions progressed to superficial skin necrosis before eventually healing, in parts leaving scars and hyperpigmentation, over the course of 2–3 weeks.

Retrospective anamnesis made the insect exposure reported the most likely cause of the skin lesions and prompted us to initiate a systematic search for beetles in the area.

All persons gave their informed consent prior to their inclusion in this report. Details that might disclose the identity of the subjects have been omitted.

### Sample collection

Before the setting up of the traps, two specimens of *Paederus balcanicus* were already collected using an aspirator at location A. Further *Paederus balcanicus* were collected with light traps: 15 specimens at location A, 13 specimens at location B, and 2 specimens at location C. No individuals of the genus *Paederus* were captured with the pitfall traps.

### Sample specification

**Fig. 2:**
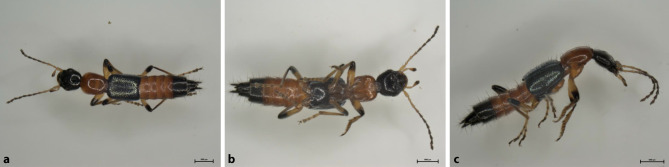
Dorsal (**a**), ventral (**b**) and lateral (**c**) view of *Paederus balcanicus*

A characteristic morphological feature of *Paederus balcanicus *is that compared to the length of the neck shield, the wing covers are longer. Furthermore, in comparison to *Paederus riparius*, the shape of the head is trapezoidal, as the long temples, which are almost twice as long as the eye diameter visible from above, narrow strongly towards the back. Punctation of the elytra is fine and sparse, with spaces between the dots clearly wider on average than the dot diameter ([8]; Fig. 2).

Moreover, morphological specification of *Paederus balcanicus* was confirmed by DNA barcoding (GenBank IDs: MZ563430; MZ563432-MZ563434). Three different haplotypes were documented. The beetles from this study cluster with *Paederus balcanicus* sequences from Austria and Montenegro at analysis in BOLD systems (PAVEA003-21 to PAVEA006-21).

## Discussion

Similar to other insects causing skin lesions, *Paederus* species have recurrently been linked to human injury including local mass breeding forcing the evacuation of entire communities [2, 10]. We describe here our observation of a cluster of persons with skin injury associated with a *Paederus* species in Austria. While the cluster was confined to a relatively small group of seven persons in this case, it highlights that raising awareness and planning of adequate measures for prevention of further and larger outbreaks of *Paederus*-associated disease are warranted in Central Europe.

We identified *Paederus balcanicus* as a potential cause of dermatitis linearis using morphological and molecular techniques. Although we did not demonstrate pederin in the hemolymph of the collected beetles, we consider the circumstantial evidence presented here as highly indicative of a relevant medical role of *P. balcanicus*. Our finding is of interest, as *P. balcanicus *has not been recognized as one of the toxic *Paederus* species so far. Little is known about this species and only few publications are available, but it has been described as stenotopic (occurring only in one or a few habitats), hygrophilous (preferring moist habitats), and paludicolous (marsh-dwelling) [11, 12]. The first observation of *P. balcanicus* in the Austrian Lake Neusiedl area dates back to 1957, when Scheerpeltz described collected rove beetles as *P. trapeziceps*, which turned out to be a synonym of *P. balcanicus* [13]. More recently, the occurrence of *P. balcanicus* in two other Austrian locations has been documented [11, 14]. Further systematic investigations are needed to better understand the biology, life cycle, behavior and distribution of *Paederus balcanicus*.

Females of the genus *Paederus* have been reported to use the toxin pederin to chemically defend themselves, their eggs and their offspring against predators [15]. Beyond that, this toxin was found to exhibit potent antitumor activity caused by selective inhibition of the eukaryotic ribosome [16]. In cytotoxicity assays using human carcinoma cell lines, IC_50_ values in the subnanomolar range were observed [17]. In the beginning, studies of the anticancer effect of pederin have been hampered by the fact that the true producer of pederin, a bacterial endosymbiont of *Paederus* beetles, is not culturable in a laboratory setting. Recently, however, the successful chemical synthesis not only of pederin itself but also of related molecules enabled the exploration of the potential of this promising family of anticancer drug candidates [18].

A number of measures to prevent linear dermatitis associated with *Paederus* species have been proposed. The most important actions are awareness-building measures in regions with resident occurrence of *Paederus* such as distribution of information on looks and behavior of the beetles, the importance of avoiding fluorescent lights to prevent attraction of the beetles at night, the correct removal of beetles from the skin to prevent toxin release, and the correct treatment of skin lesions [5]. In our case, the authors recommend the instalment of information and warning signs in the affected public recreational area of Lake Neusiedl.

In conclusion, we report for the first time (a) an outbreak of dermatitis linearis associated with exposure to an autochthonous *Paederus* species in Austria, and (b) that contact to the species *Paederus balcanicus* may be a cause of dermatitis linearis in humans. We consider dermatitis linearis outbreaks due to *Paederus *occurrence an emerging public health concern.
